# RNA Interference of GADD153 Protects Photoreceptors from Endoplasmic Reticulum Stress-Mediated Apoptosis after Retinal Detachment

**DOI:** 10.1371/journal.pone.0059339

**Published:** 2013-03-29

**Authors:** Hong Zhu, Jin Qian, Wenqiu Wang, Quan Yan, Ying Xu, Yuan Jiang, Lei Zhang, Fengqing Lu, Weiting Hu, Xi Zhang, Fenghua Wang, Xiaodong Sun

**Affiliations:** 1 Department of Ophthalmology, Shanghai First People’s Hospital, School of Medicine, Shanghai JiaoTong University, Eye Research Institute of Shanghai JiaoTong University, Shanghai, China; 2 Department of Ophthalmology, No.3 People’s Hospital Affiliated to Shanghai JiaoTong University School of Medicine, Shanghai, China; 3 Eye Research Institute of Shanghai JiaoTong University, Shanghai, China; 4 Shanghai Key Laboratory of Fundus Disease, Shanghai, China; Innsbruck Medical University, Austria

## Abstract

**Background:**

Apoptosis of photoreceptors plays a critical role in the vision loss caused by retinal detachment (RD). Pharmacologic inhibition of photoreceptor cell death may prevent RD. This study investigated the role of GADD153 that participates in endoplasmic reticulum (ER) stress-mediated apoptosis of photoreceptor cells after RD.

**Methods:**

Retinal detachment was created in Wistar rats by subretinal injection of hyaluronic acid. The rats were then randomly divided into four groups: normal control group, RD group, GADD153 RNAi group and vehicle group. RNA interference of GADD153 was performed using short hairpin RNA (shRNA). Expressions of GADD153 mRNA and protein were examined by RT-PCR and Western blotting analysis, respectively. GADD153 protein distribution in the retinal cells was observed using immunofluorescence confocal laser scanning microscopy. Apoptosis of retinal cells was determined by TdT-mediated fluorescein-16-dUTP nick-end labeling (TUNEL) assay.

**Results:**

Lentivirus GADD153 shRNA with the most effective silencing effect was chosen for *in vivo* animal study and was successfully delivered into the retinal tissues. GADD153 mRNA and protein expressions in GADD153 RNAi group were significantly lower than those in the RD group. Silencing of GADD153 by RNAi protected photoreceptors from ER stress-induced apoptosis.

**Conclusion:**

ER stress-mediated pathway is involved in photoreceptor cell apoptosis after RD. GADD153 is a key regulatory molecule regulating ER-stress pathways and plays a crucial role in the apoptosis of photoreceptor cells after RD.

## Introduction

Acute or chronic detachment of the retina from the retinal pigment epithelium (RPE) surface is the leading cause of vision loss in patients with diabetic retinopathy, pathological myopia, posterior eye trauma and age related macular degeneration. Retinal detachment (RD) results in not only the separation of the photoreceptor cell layer from the apical surface of the RPE but also the expansion of the interphotoreceptor space. Photoreceptor cell death by apoptosis, which could be observed immediately after RD, plays a critical role in visual loss. Therefore, new insights into the mechanisms underlying photoreceptor cell apoptosis in RD would be of clinical interest and could lead to new treatments.

Growth arrest DNA damage-inducible gene 153 (GADD153), also known as C/EBP homologous protein, plays a vital role in ER stress-induced apoptosis. It has been proven to be involved in the pathogenesis of various diseases, including diabetes [1], brain ischemia [2], [3] and neurodegenerative disease [4]. Over expression of GADD153 and microinjection of GADD153 protein have been reported to cause cell cycle arrest and/or apoptosis [5]–[8]. Previously we found that the expression of GADD153 was temporally and spatially associated with the apoptosis of photoreceptor cells, suggesting the involvement of ER stress-mediated pathway in the apoptosis of photoreceptor cell after RD [9]. Recently, researchers found CHOP−/− mice exhibited reduced apoptosis in response to ER stress and GADD153-deficient cells were resistant to ER stress-induced apoptosis [10], [11]. Considering that GADD153 has been found as a key molecule in ER stress pathway, it would be of interest to know whether interference of GADD153 could protect photoreceptor from apoptosis in RD.

In order to further confirm whether GADD153 participates in ER stress- mediated apoptosis of photoreceptor cells after RD, in this study, we suppressed GADD153 expression by injecting lentivirus GADD153 shRNA into the subretinal space, and observed the apoptosis of photoreceptor cells after RD.

## Materials and Methods

### Generation of Lentivirus GADD153 shRNA

Lentiviral vectors encoding shRNAs against GADD153, or lentiviral vectors without encoding GADD153 shRNA were synthesized by Telebio Biomedical Co., Ltd (Shanghai, China). Vector particles were prepared by Lentivirus Expression Systems. Three constructed lentivirus shRNAs targeting different sites of GADD153 and a negative control lentivirus shRNA were transduced in HEK 293T cells (CRL11268, American Type Culture Collection, Rockville, MD) to test the efficacy [12], [13] (Table. S1). The lentivirus GADD153 shRNA (LV-GADD153-sh) with the best silencing efficacy was selected for *in vivo* experimental RD study. (Figure S1, Figure S2).

### Animals and Experimental RD

All the experiments were humanely performed in accordance with the Statement of Association for Research in Vision and Ophthalmology for the Use of Animals in Ophthalmic and Vision Research, and the protocols were approved by the Shanghai First People’s hospital institutional review board. A total of 124 male Wistar rats (weighing 180–220 g, 7–8 weeks old) were supplied by the Laboratory Animal Center of the institute, and were divided into four groups: normal control group (*n* = 4), RD group (*n* = 40), LV-GADD153-sh+RD group (*n* = 40), and vehicle (Lentiviral vector) +RD group (*n* = 40).

Retinal detachment was created by subretinal injection of 10 mg/ml sodium hyaluronate (Bausch & Lomb Freda, China) as described previously [14]–[17]. Briefly, the rats were anesthetized with an intraperitoneal (i.p.) injection of 10% chloral hydrate; a 30-gauge needle was inserted into the subretinal space via an external trans-scleral trans-choroidal approach. 1% sodium hyaluronate was gently injected into the subretinal space to enlarge the retinal detachment (50 µl each makes 50% RD) [18].

### In vivo Delivery of Vectors

To deliver the vectors into the eye, the animals were anesthetized with an i.p. injection of 10% chloral hydrate. Subretinal injection was performed under a microscopeand under sterile conditions according to the method reported previously [19]. In brief, a small incision was made in the sclera at approximately 1.5 mm posterior to the limbus with a 30-gauge needle. The needle was slowly introduced into the vitreous cavity, then to subretinal space by producing a small hole in the nasal peripheral retina. A single 5 µl vector suspension containing 10^8^ TU/ml of LV-GADD153-sh or lentiviral vector was administered in 30 s. Two weeks after delivering the vectors, RD was created as described above.

### RT-PCR Analysis

Two weeks after delivering the vectors, samples of rat retina collected at the time points of day1, 2, 4, and 7 after RD were quickly frozen in liquid nitrogen. Total RNA was isolated using Trizol (Invitrogen, Carlsbad, CA, USA). cDNA was synthesized from 2 µg total RNA in 20 µl reaction mixture using a RT-PCR kit (Invitrogen, Carlsbad, CA, USA) according to the manufacturer’s protocol. Samples of cDNA were subjected to GADD153 amplification with β-actin used as a housekeeping gene. The primers used were as following: GADD153, 5′ATGGCAGCTGAGTCTCTGCC3′ and 5′TGATGGTGCTGGGTACACTCC3′. Semi-quantitative analysis was carried out by measuring the intensity abundance of PCR products in gel photographs using Gene Genius Image software.

### Western Blotting Analysis

Two weeks after delivering the vectors, samples were collected on 1, 2, 4 and 7 days after RD. Samples were run on 4% to 12% SDS polyacrylamidegel electrophoresis and transferred onto nitrocellulose membranes (Whatman, Maidstone, UK). After being blocked with 3% nonfat dried milk, the membrane was reacted with GADD153 (1∶100; Santa Cruz Biotechnology, Santa Cruz, CA). The density of the signal was quantified using Bandscan43 software and the protein expression levels were normalized for β-actin (1:50000; Sigma-Aldrich Chemical Co., St Louis, Mo, USA).

### Immunofluorescence Examination

GADD 153 immunofluorescence examination was performed on sections obtained from the retina. Sections were treated with 0.5 mg/ml trypsin and each section was incubated for 30 min in phosphate-buffered saline (PBS) containing 5% skim milk to block non-specific binding, followed by incubation with antibodies against GADD153 (1∶50, Santa Cruz Biotechnology, Santa Cruz, CA) overnight at 4°C. Sections were then incubated with Envision goat-anti-rabbit IgG HRP polymer (Dako, Carpinteria, CA) for 1 h at room temperature. Signals were amplified with TSA (tyramide signal amplification) biotin system (PerkinElmer, Boston, MA). The sections were stained with propidium iodine (PI) for 15 min to illustrate retinal cell distribution and to clear retinal layers such as outer nuclear layer (ONL), inner nuclear layer (INL) and ganglion cell layer (GCL). Laser-scanning confocal microscopy was used to observe and count the number of GADD153-positive cells and PI stained cells in ONL at 1.0–1.5 mm from the optic disc. The percentages of GADD153 positive cells/PI stained cells were calculated by LSM 510 Expert Mode SP2 software.

### Assessment of Retinal Outer Nuclear Layer Damage

The eyes (*n* = 3) were enucleated one week after RD and were embedded in paraffin. The eye wall sections were then stained with hematoxylin and eosin (H&E). The retinal histoarchitecture was evaluated by light microscopy. The ONL thickness was determined by Image-Pro Plus software on day 7 after experimental RD. Five sections were selected from the same quadrant in each eye. The thickness was measured at 10 points in each section by independent observers.

### TdT-mediated Fluorescein-16-dUTP Nick-end Labelling (TUNEL) Assay

Enucleated eyes were immediately fixed with 10% formalin and embedded in paraffin. Transverse sections (5 µm) in the nasal retina were prepared. TUNEL assay was peformed to detect the apoptotic cells using the Apoptosis Detection System, Fluorescein (Promega, Madison, WI, USA). The cell nuclei were revealed by staining retinal sections with PI (1∶3000, Molecular Probes, Eugene, OR, USA). Laser-scanning confocal microscopy (LSM 510, Zeiss, Jena, Germany) was employed to observe and count the number of TUNEL-positive cells and PI stained cells in ONL. The percentages of TUNEL positive cells and PI stained cells in ONL were calculated by LSM 510 Expert Mode SP2 software (Zeiss, Jena, Germany).

### Statistical Analysis

Statistical analysis was performed using SPSS (SPSS 13.0, SPSS Institute Inc., Chicago, IL, USA). The results were presented as means ± SD. The One-Sample Kolmogorov-Smirnov test was used to measure whether these data were in a normal distribution. Statistical significance was calculated with a one factor ANOVA test. A *P* value less than 0.05 was considered statistically significant.

## Results

### GADD153 RNA Interference Resulted in Decreased GADD153 mRNA and Protein Contents in vivo

The experimental RD was induced two weeks after subretinal injection of the LV- GADD153-sh or vectors 5×10^5^ TU. We firstly examined GADD153 mRNA expressions from whole retina at 1 day, 2 day, 4 day and 7 day after RD by using RT-PCR. The GADD153 mRNA was hardly found expressed in normal control retinal tissues. It increased as early as 1 day after experimental RD. The expressions of GADD153 mRNA in RNAi group significantly decreased at different time points after RD compared with those in the RD group and vehicle group.

**Figure 1:**
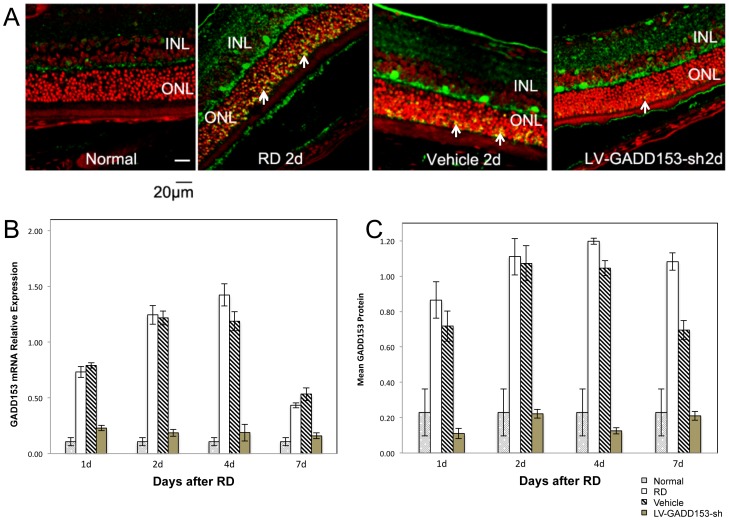
GADD153 RNA interference decreased GADD153 mRNA and protein contents in vivo. (A) Representative retinal sections from rats on 2 days after RD. Nuclei were counterstained with PI (*red*). GADD153 staining (*green*) is evident in ONL. Because of the digital overlay of PI and GADD153 staining in Figure 1, the location of the GADD153 staining should be in yellow color in the outer segments. The yellow color were observed in the ONL 2 days after detachment and disappeared under GADD153 RNAi. There were no GADD153-positive cells in retinas of the normal control group. GADD153-positive cells in ONL increased after retinal detachment, most GADD153-positive cells were located in the ONL layer (GADD153 distribution was shown with white arrows). Compared with retinal detachment group, GADD153-positive cells decreased in RNA interference group (LV-GADD153-sh) but not in Vehicle group. (B) RT-PCR semi- quantification of GADD153 mRNA expression in RD rats under shRNA, vehicle or non-treatment. GADD153 mRNA increased in RD groups with the highest expression found at 2 to 4 days after detachment and then decreased. The change of mRNA in vehicle group was the same as that in the non-treated RD group. The RNA interference group (LV-GADD153-sh) showed a very low level of GADD mRNA. (C) Signal intensities of GADD153 protein were measured by Bandscan analysis and the ratios of GADD153 protein expression to β-actin in each sample were given. West blotting revealed similar changes of protein expression at different time points after RD. The RNA interference group (LV-GADD153-sh) showed a very low level of GADD protein.

Temporal observation of GADD153 expression using Western blotting revealed an increase of GADD153 protein level, and immunofluorescence microscopy demonstrated that positive staining was mainly located in the nucleus and confined only to the ONL at different time points after RD (Figure 1). The expressions of GADD153 protein in LV-GADD153-shRNA group significantly decreased compared with those in the RD group (*P*<0.05), while the GADD153 protein expressions in the vehicle group showed no significant decrease.

### GADD153 RNA Interference Decreased ONL Damage and Protected Retina

**Figure 2:**
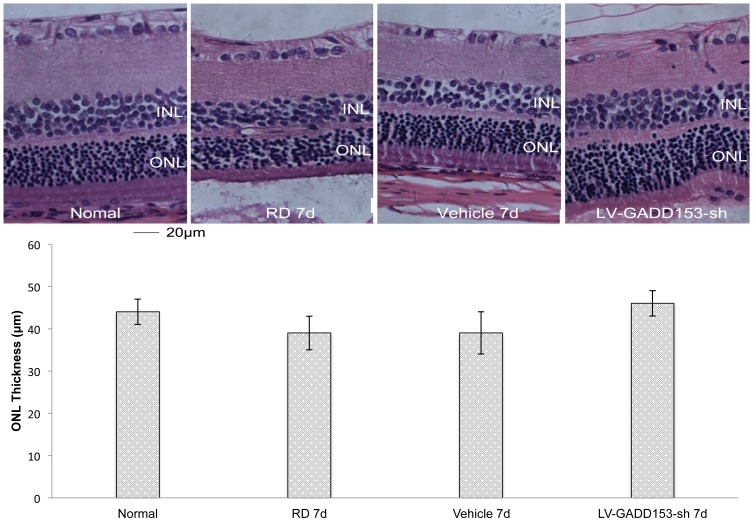
Analysis of ONL thickness in the retina after RD. (A) Representative histopathological images of retina section from normal rat eyes and rat eyes treated with GADD153 RNA interference (LV-GADD 153-sh), vehicle, and non-treated (RD) after 7 days of experimental RD. (B) In all cases, treatment with LV-GADD 153-sh led to better preservation of ONL thickness than RD groups.

In RD rats, ONL thickness of retina rapidly and significantly decreased after 7 days. The ONL structure changed slightly. Thickness of layers below the outer plexiform layer were never altered after RD. The ONL of retinas exposed to LV-GADD153-sh was substantially thicker than that from control eyes injected with LV vectors or untreated RD eyes. After 7 days of RD, the average thickness of ONL preservation of eyes that received LV-GADD153-sh was 46±3 µm compared with 39±5 µm in the eyes that received LV-vector or 39±4 µm in untreated RD eyes (ANOVA; *P*<0.001; Figure 2).

### GADD153 RNA Interference Decreased TUNEL-positive Photoreceptor Cells and Protected Retina

**Figure 3:**
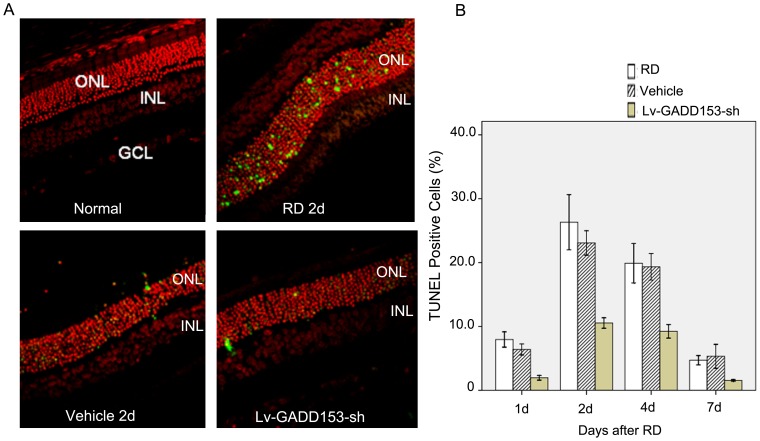
GADD153 RNA interference decreased TUNEL cell counts after RD in vivo. (A) Representative retinal sections from rats on 2 days after RD. No TUNEL-positive cells were observed in normal control group. The TUNEL-positive cells in ONL increased after retinal detachment. Most TUNEL-positive cells were located in the ONL layer. Compared with retinal detachment group, TUNEL-positive cells decreased in RNA interference group (LV-GADD153-sh) but not in vehicle group. (B) TUNEL-positive cells in ONL increased first and then decreased after retinal detachment, with the highest expression found at 2 days after detachment and the lowest at 7 days after detachment. Compared with retinal detachment group, TUNEL-positive cells decreased at all time points after retinal detachment in GADD153 RNAi group.

We assessed photoreceptor death after RD using TUNEL staining, which detects DNA fragmentation in apoptotic or necrotic nuclei. Hardly any TUNEL-positive cells were found in the normal control group. In the RD group, TUNEL-positive photoreceptor cells appeared on day 1 (7.96%). The number of TUNEL-positive photoreceptor cells peaked on day 2 (20.32%) and day 4 (19.9%), and then decreased on day 7 (4.72%). In GADD153 RNAi group, the number of TUNEL-positive photoreceptor cells also peaked from day 2 (10.53%) to day 4 (9.24%), then attenuated to 1.54% on day 7. Compared with the RD group, the apoptotic cells in GADD153 RNAi group significantly decreased at different time points after RD. The injection of vehicle did not reduce the number of TUNEL-positive cells in ONL at all time points (Figure 3).

## Discussion

Our previous study showed that GADD153 was present in the retina of an RD model, accompanied by apoptosis of photoreceptor cells [9].In this study we observed that silencing GADD153 using RNAi technology could inhibit the apoptosis of photoreceptor cells and protect retina in experimental RD. These findings strongly suggested that the ER stress pathway activated by GADD153 was involved in the apoptosis of photoreceptor cells after RD.

Expression of GADD153 was at a very low level in the normal retina of rats, and it increased on day 2 and day 4 after RD, which was consistent with the peak of photoreceptor cell apoptosis. GADD153-positive cells were confined to the ONL, where most apoptotic cells could also be found, and this was consistent with the result of our pervious study [9]. In the present study, we targeted GADD153 expression using RNAi technology (Table S1). We induced RD model after two weeks of Lentivirus injection. It is in agreement with the reported transfection time of lentivirus in retinal pigment epithelium [20]. In our pretest experiment, the greatest knockdown effect was achieved with the use of current LV-GADD153-shRNA sequence, and it demonstrated the suppression of GADD153 levels by 70% in vitro. In the present in vivo study, we were able to safely deliver lentivirus vector to the subretinal space in a rat model, with no apparent adverse effects caused by either the viral vector or the procedure itself. We were also able to show that the vector successfully delivered the gene product within the retina using RT-PCR and west blotting.

GADD153 positive cells and apoptotic photoreceptor cells were observed simultaneously on day 1. The number of GADD153 positive cells and apoptotic photoreceptor cells peaked on day 2 to day 4, and then decreased on day 7 in RD group, vehicle group and LV-GADD153-sh group. The number of apoptotic photoreceptor cells and the expression of GADD153 in GADD153 RNAi group significantly decreased at different time points after RD compared with those in RD group. The number of GADD153 positive cells and apoptotic cells in ONL both decreased in GADD153 RNAi group. These results suggested that the decrease of retinal GADD153 expression was caused by the specific silencing effect of GADD153 shRNA, leading to inhibition of photoreceptor cell apoptosis to a great extent. It is indicated that after RD, the expression of GADD153 increased in the photoreceptor cells, which then caused cell cycle arrest and DNA damage, finally leading to the cell apoptosis. When GADD153 expression was inhibited, the ER stress pathway was widely blocked, possibly through GADD153 targeting the downstream molecules such as TRB3, GADD34, and finally attenuated the apoptosis. These suggest that ER stress-mediated pathway participated in the apoptosis of photoreceptor cells after RD. To evaluate the neuroprotection of GADD153 RNAi against RD-induced cell death, we measured the thickness of the ONL. There are several methods available to measure the ONL thickness, such as the ONL thickness normalized to retinal thickness [21]–[24], measurement of the ONL thickness from the optic disc at the same intervals on the photographs [25], measurement along four different meridians to make color-coded topographic maps [26] and acquirement an average from directly measurement in each H&E staining retina section [27]–[31]. We chose to measure ONL thickness in the sections under H&E staining because it is also a method frequently used in retinal studies. Our data showed the ONL is 46±3 µm and 44.03±3 µm, respectively, in LV-GADD153-shRNA treated group and in normal group 7 days after detachment. There is no significant difference between the ONL thicknesses in normal and LV-GADD153-shRNA treated eyes. However, the ONL thicknesses in both of the above two groups are thicker than that in the RD control eyes (39±4 µm, *P*<0.001, Figure 2). This trend indicated the protective effect of GADD153 RNAi in experiment retinal detachment. GADD153, as a key marker for ER stress, plays a crucial role in photoreceptor cell apoptosis after RD.

Interestingly, there were reports showing that some agents can protect retina from the damage after RD without interfering GADD153 expression, which indicates that multi-pathways take part in retinal damage after RD. In this study, we found that shRNA to GADD153 dramatically reduced photoreceptor cells apoptosis after RD. These findings encouraged us to hypothesize that GADD153 RNAi could have a protective role by blocking ER stress-induced photoreceptor cell death. However, whether it takes part in other pathway of photoreceptor apoptosis is still unclear. Considering that ER stress-induced apoptosis is a critical step in the pathogenesis of many neurodegenerative diseases, silencing GADD153 can function as a survival factor for neurons [32]–[36]. Chen et al showed that CHOP partially mediated ER stress-induced neuronal death and suggested that suppression of CHOP activation may contribute to BDNF-mediated neuroprotection during ER stress responses [37]. In another case, it was found that CHOP knockdown prevented perturbations in the AKT/FOXO3a pathway in response to ER stress-induced apoptosis in neuronal cells [38].

In summary, this study demonstrates that ER stress-mediated pathway should be considered as a mechanism of inducing apoptosis in photoreceptor cells after RD. GADD153 is the key regulatory molecule regulating the ER Stress pathway, thus plays a crucial role in the photoreceptor cell apoptosis after RD. The findings in the present study may cast new lights on the therapeutic strategies aiming at rehabilitation and reconstruction of visual function after RD, as photoreceptor cells may be protected from RD-induced damage by subretinal injection of GADD153 RNAi. Gene therapy might be a good adjuvant to present treatments for complex types of RD.

## Supporting Information

Figure S1 **Delivery efficiency of lentivirus GADD153 shRNA-1 in vivo under stereo fluorescence microscope.** Expression of GFP was firstly observed 1 week after the injection of lentivirus GADD153 shRNA-1 (A) and peaked at 2 weeks (B). The number of GFP-positive cells reached the peak at 2 weeks, which is the time point showing the highest transduction efficiency. Therefore, this time point was chosen to make the RD model.
(DOC)

Figure S2 **Delivery efficiency of lentivirus GADD153 shRNA-1 in vitro under fluorescence microscope.** Expression of GFP was firstly observed 1 week after the injection of lentivirus GADD153 shRNA-1 (A), peaked at 2 weeks (B) in retina frozen sections.
(DOC)

Table S1 **Three alternative sequences of GADD153 shRNA and the control construction.** All the alternative sequences of GADD153 shRNA and the control construction are shown.
(DOC)

## References

[1] Wang J, Takeuchi T, Tanaka S, Kubo SK, Kayo T, et al (1999) A mutation in the insulin 2 gene induces diabetes with severe pancreatic beta-cell dysfunction in the Mody mouse. J Clin Invest 103: 27–37. 9884331 10.1172/JCI4431 PMC407861

[2] Kumar R, Azam S, Sullivan JM, Owen C, Cavener DR, et al (2001) Brain ischemia and reperfusion activates the eukaryotic initiation factor 2 alpha kinase PERK. J Neurochem 77: 1418–1421. 11389192 10.1046/j.1471-4159.2001.00387.x

[3] Paschen W, Gissel C, Linden T, Althausen S, Doutheil J (1998) Activation of gadd153 expression through transient cerebral ischemia: evidence that ischemia causes endoplasmic reticulum dysfunction. Brain Res Mol Brain Res 60: 115–122. 9748529 10.1016/s0169-328x(98)00180-6

[4] Ryu EJ, Harding HP, Angelastro JM, Vitolo OV, Ron D, et al (2002) Endoplasmic reticulum stress and the unfolded protein response in cellular models of Parkinson’s disease. J Neurosci 22: 10690–10698. 12486162 10.1523/JNEUROSCI.22-24-10690.2002 PMC6758450

[5] Matsumoto M, Minami M, Takeda K, Sakao Y, Akira S (1996) Ectopic expression of CHOP (GADD153) induces apoptosis in M1 myeloblastic leukemia cells. FEBS Lett 395: 143–147. 8898082 10.1016/0014-5793(96)01016-2

[6] Maytin EV, Ubeda M, Lin JC, Habener JF (2001) Stress-inducible transcription factor CHOP/gadd153 induces apoptosis in mammalian cells via p38 kinase-dependent and -independent mechanisms. Exp Cell Res 267: 193–204. 11426938 10.1006/excr.2001.5248

[7] Barone MV, Crozat A, Tabaee A, Philipson L, Ron D (1994) CHOP (GADD153) and its oncogenic variant, TLS-CHOP, have opposing effects on the induction of G1/S arrest. Genes Dev 8: 453–464. 8125258 10.1101/gad.8.4.453

[8] Gotoh T, Oyadomari S, Mori K, Mori M (2002) Nitric oxide-induced apoptosis in RAW 264.7 macrophages is mediated by endoplasmic reticulum stress pathway involving ATF6 and CHOP. J Biol Chem 277: 12343–12350. 11805088 10.1074/jbc.M107988200

[9] Liu H, Qian J, Wang F, Sun X, Xu X, et al (2010) Expression of two endoplasmic reticulum stress markers, GRP78 and GADD153, in rat retinal detachment model and its implication. Eye (Lond) 24: 137–144. 19218986 10.1038/eye.2009.20

[10] Oyadomari S, Koizumi A, Takeda K, Gotoh T, Akira S, et al (2002) Targeted disruption of the Chop gene delays endoplasmic reticulum stress-mediated diabetes. J Clin Invest 109: 525–532. 11854325 10.1172/JCI14550 PMC150879

[11] Zinszner H, Kuroda M, Wang X, Batchvarova N, Lightfoot RT, et al (1998) CHOP is implicated in programmed cell death in response to impaired function of the endoplasmic reticulum. Genes Dev 12: 982–995. 9531536 10.1101/gad.12.7.982 PMC316680

[12] An DS, Xie Y, Mao SH, Morizono K, Kung SK, et al (2003) Efficient lentiviral vectors for short hairpin RNA delivery into human cells. Hum Gene Ther 14: 1207–1212. 12908971 10.1089/104303403322168037

[13] Akimana C, Al-Khodor S, Abu Kwaik Y (2010) Host factors required for modulation of phagosome biogenesis and proliferation of Francisella tularensis within the cytosol. PLoS One 5: e11025. 20552012 10.1371/journal.pone.0011025 PMC2883998

[14] Wang XZ, Ron D (1996) Stress-induced phosphorylation and activation of the transcription factor CHOP (GADD153) by p38 MAP Kinase. Science 272: 1347–1349. 8650547 10.1126/science.272.5266.1347

[15] Oyadomari S, Takeda K, Takiguchi M, Gotoh T, Matsumoto M, et al (2001) Nitric oxide-induced apoptosis in pancreatic beta cells is mediated by the endoplasmic reticulum stress pathway. Proc Natl Acad Sci USA 98: 10845–10850. 11526215 10.1073/pnas.191207498 PMC58562

[16] Nakazawa T, Matsubara A, Noda K, Hisatomi T, She H, et al (2006) Characterization of cytokine responses to retinal detachment in rats. Mol Vis 12: 867–878. 16917487

[17] Zacks DN, Hanninen V, Pantcheva M, Ezra E, Grosskreutz C, et al (2003) Caspase activation in an experimental model of retinal detachment. Invest Ophthalmol Vis Sci 44: 1262–1267. 12601057 10.1167/iovs.02-0492

[18] Hisatomi T, Sakamoto T, Murata T, Yamanaka I, Oshima Y, et al (2001) Relocalization of Apoptosis-Inducing Factor in Photoreceptor Apoptosis Induced by Retinal Detachment in Vivo. Am J Pathol 158: 1271–1278. 11290545 10.1016/S0002-9440(10)64078-3 PMC1891920

[19] Lei B, Zhang K, Yue Y, Ghosh A, Duan D (2009) Adeno-associated virus serotype-9 efficiently transduces the retinal outer plexiform layer. Mol Vis 15: 1374–1382. 19626133 PMC2713732

[20] Murakami Y, Ikeda Y, Yonemitsu Y, Onimaru M, Nakagawa K, et al (2008) Inhibition of nuclear translocation of apoptosis-inducing factor is an essential mechanism of the neuroprotective activity of pigment epithelium-derived factor in a rat model of retinal degeneration. Am J Pathol 173: 1326–1338. 18845835 10.2353/ajpath.2008.080466 PMC2570123

[21] Roh MI, Murakami Y, Thanos A, Vavvas DG, Miller JW (2011) Edaravone, an ROS scavenger, ameliorates photoreceptor cell death after experimental retinal detachment. Invest Ophthalmol Vis Sci 52: 3825–3831. 21310909 10.1167/iovs.10-6797 PMC3109058

[22] Besirli CG, Chinskey ND, Zheng QD, Zacks DN (2010) Inhibition of retinal detachment-induced apoptosis in photoreceptors by a small peptide inhibitor of the fas receptor. Invest Ophthalmol Vis Sci 51: 2177–2184. 19850829 10.1167/iovs.09-4439 PMC2868404

[23] Zacks DN, Boehlke C, Richards AL, Zheng QD (2007) Role of the Fas-signaling pathway in photoreceptor neuroprotection. Arch Ophthalmol 125: 1389–1395. 17923548 10.1001/archopht.125.10.1389

[24] Kayama M, Nakazawa T, Thanos A, Morizane Y, Murakami Y, et al (2011) Heat shock protein 70 (HSP70) is critical for the photoreceptor stress response after retinal detachment via modulating anti-apoptotic Akt kinase. Am J Pathol 178: 1080–1091. 21356360 10.1016/j.ajpath.2010.11.072 PMC3069883

[25] Shimazaki H, Hironaka K, Fujisawa T, Tsuruma K, Tozuka Y, et al (2011) Edaravone-loaded liposome eyedrops protect against light-induced retinal damage in mice. Invest Ophthalmol Vis Sci 52: 7289–7297. 21849425 10.1167/iovs.11-7983

[26] Tanito M, Kaidzu S, Ohira A, Anderson RE (2008) Topography of retinal damage in light-exposed albino rats. Exp Eye Res 87: 292–295. 18586030 10.1016/j.exer.2008.06.002

[27] Shyong MP, Lee FL, Hen WH, Kuo PC, Wu AC, et al (2008) Viral delivery of heme oxygenase-1 attenuates photoreceptor apoptosis in an experimental model of retinal detachment. Vision Res 48: 2394–2402. 18713643 10.1016/j.visres.2008.07.017

[28] Xie Z, Chen F, Wu X, Zhuang C, Zhu J, et al (2012) Safety and efficacy of intravitreal injection of recombinant erythropoietin for protection of photoreceptor cells in a rat model of retinal detachment. Eye (Lond) 26: 144–152. 22020175 10.1038/eye.2011.254 PMC3259587

[29] Mockel A, Obringer C, Hakvoort TB, Seeliger M, Lamers WH, et al (2012) Pharmacological modulation of the retinal unfolded protein response in Bardet-Biedl syndrome reduces apoptosis and preserves light detection ability. J Biol Chem 287: 37483–37494. 22869374 10.1074/jbc.M112.386821 PMC3481343

[30] Eigeldinger-Berthou S, Meier C, Zulliger R, Lecaudé S, Enzmann V, et al (2012) Rasagiline interferes with neurodegeneration in the Prph2/rds mouse. Retina 32: 617–628. 21878836 10.1097/IAE.0b013e31821e2070

[31] Lewis GP, Chapin EA, Byun J, Luna G, Sherris D, et al (2009) Muller cell reactivity and photoreceptor cell death are reduced after experimental retinal detachment using an inhibitor of the Akt/mTOR pathway. Invest Ophthalmol Vis Sci 50: 4429–4435. 19369237 10.1167/iovs.09-3445

[32] McCullough KD, Martindale JL, Klotz LO, Aw TY, Holbrook NJ (2001) Gadd153 sensitizes cells to endoplasmic reticulum stress by down-regulating Bcl2 and perturbing the cellular redox state. Mol Cell Biol 21: 1249–1259. 11158311 10.1128/MCB.21.4.1249-1259.2001 PMC99578

[33] Puthalakath H, O’Reilly LA, Gunn P, Lee L, Kelly PN, et al (2007) ER stress triggers apoptosis by activating BH3-only protein Bim. Cell 129: 1337–1349. 17604722 10.1016/j.cell.2007.04.027

[34] Ruiz-Vela A, Opferman JT, Cheng EH, Korsmeyer SJ (2005) Proapoptotic BAX and BAK control multiple initiator caspases. EMBO Rep 6: 379–385. 15776018 10.1038/sj.embor.7400375 PMC1299285

[35] Wei MC, Zong WX, Cheng EH, Lindsten T, Panoutsakopoulou V, et al (2001) Proapoptotic BAX and BAK: a requisite gateway to mitochondrial dysfunction and death. Science 292: 727–730. 11326099 10.1126/science.1059108 PMC3049805

[36] Zhang D, Armstrong JS (2007) Bax and the mitochondrial permeability transition cooperate in the release of cytochrome c during endoplasmic reticulum-stress-induced apoptosis. Cell Death Differ 14: 703–715. 17170750 10.1038/sj.cdd.4402072

[37] Chen G, Fan Z, Wang X, Ma C, Bower KA, et al (2007) Brain-derived neurotrophic factor suppresses tunicamycin-induced upregulation of CHOP in neurons. J Neurosci Res 85: 1674–1684. 17455323 10.1002/jnr.21292 PMC3085896

[38] Ghosh AP, Klocke BJ, Ballestas ME, Roth KA. (2012) CHOP potentially co-operates with FOXO3a in neuronal cells to regulate PUMA and BIM expression in response to ER stress. PLoS One 7: e39586. Epub 2012 Jun 28. 10.1371/journal.pone.0039586 PMC3386252 22761832

