# Supplementary material for: RNA Interference of GADD153 Protects Photoreceptors from Endoplasmic Reticulum Stress-Mediated Apoptosis after Retinal Detachment
Source: PLoS One. 2013 Mar 29;8(3):e59339. doi: 10.1371/journal.pone.0059339 (PMC3612068; doi:10.1371/journal.pone.0059339)

Figure S1. Delivery efficiency of lentivirus GADD153 shRNA-1 *in vivo* under stereo fluorescence microscope.

Expression of GFP was firstly observed 1 week after the injection of lentivirus GADD153 shRNA-1 (A) and peaked at 2 weeks (B). The number of GFP-positive cells reached the peak at 2 weeks , which is the time point showing the highest transduction efficiency. Therefore, this time point was chosen to make the RD model.


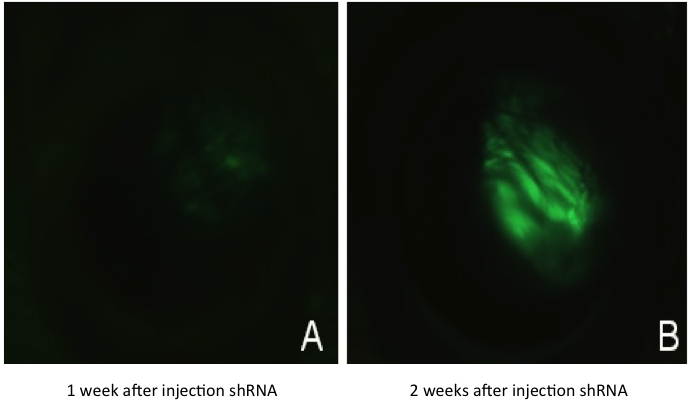

Supplement: Figure S1 — Delivery efficiency of lentivirus GADD153 shRNA-1 in vivo under stereo fluorescence microscope. Expression of GFP was firstly observed 1 week after the injection of lentivirus GADD153 shRNA-1 (A) and peaked at 2 weeks (B). The number of GFP-positive cells reached the peak at 2 weeks, which is the time point showing the highest transduction efficiency. Therefore, this time point was chosen to make the RD model. (DOC) [file pone.0059339.s001.doc]
