# Supplementary material for: RNA Interference of GADD153 Protects Photoreceptors from Endoplasmic Reticulum Stress-Mediated Apoptosis after Retinal Detachment
Source: PLoS One. 2013 Mar 29;8(3):e59339. doi: 10.1371/journal.pone.0059339 (PMC3612068; doi:10.1371/journal.pone.0059339)

Figure S2. Delivery efficiency of lentivirus GADD153 shRNA-1 *in vitro* under fluorescence microscope

Expression of GFP was firstly observed 1 week after the injection of lentivirus GADD153 shRNA-1 (A), peaked at 2 weeks (B) in retina frozen sections.


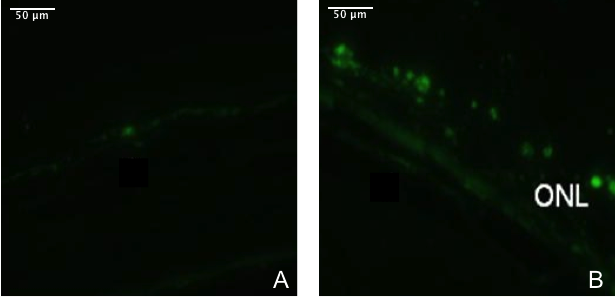

Supplement: Figure S2 — Delivery efficiency of lentivirus GADD153 shRNA-1 in vitro under fluorescence microscope. Expression of GFP was firstly observed 1 week after the injection of lentivirus GADD153 shRNA-1 (A), peaked at 2 weeks (B) in retina frozen sections. (DOC) [file pone.0059339.s002.doc]
