# Supplementary material for: RNA Interference of GADD153 Protects Photoreceptors from Endoplasmic Reticulum Stress-Mediated Apoptosis after Retinal Detachment
Source: PLoS One. 2013 Mar 29;8(3):e59339. doi: 10.1371/journal.pone.0059339 (PMC3612068; doi:10.1371/journal.pone.0059339)
Supplement: Table S1 — Three alternative sequences of GADD153 shRNA and the control construction. All the alternative sequences of GADD153 shRNA and the control construction are shown. (DOC) [file pone.0059339.s003.doc]

Table S1. Three alternative sequences of GADD153 shRNA and the control construction.

All the alternative sequences of GADD153 shRNA and the control construction are shown.

| Hairpin Name | Sequence |
| --- | --- |
| GADD-S1 | Forward: 5’-GATCCCGCGAGCGGCTCAACGAGGAATTGATATCCGTTCCTCGTTGAGCCGCTCGTTTTTTCCAAC-3’ |
|  | Reverse: 5’-GGCGCTCGCCGAGTTGCTCCTTAACTATAGGCAAGGAGCAACTCGGCGAGCAAAAAAGGTTGAGCT-3’ |
| GADD-S2 | Forward: 5’-GATCCCGCCAATTACAGTCATGGCAGTTGATATCCGCTGCCATGACTGTAATTGGTTTTTTCCAAC-3’ |
|  | Reverse: 5’-GGCGGTTAATGTCAGTACCGTCAACTATAGGCGACGGTACTGACATTAACCAAAAAAGGTTGAGCT-3’ |
| GADD-S3 | Forward: 5’-GATCCCGGAACCTGAGGAGAGAGAAATTGATATCCGTTTCTCTCTCCTCAGGTTCTTTTTTCCAAC-3’ |
|  | Reverse: 5’-GGCCTTGGACTCCTCTCTCTTTAACTATAGGCAAAGAGAGAGGAGTCCAAGAAAAAAGGTTGAGCT-3’ |
| Negative control | Forward: 5’-GATCCC(G)TTCTCCGAACGTGTCACGTTTGATATCCG ACGTGACACGTTCGGAGAA TTTTTTCCAAC-3’ |
|  | Reverse: 5’-GG(C)AAGAGGCTTGCACAGTGCAAACTATAGGCTGCACTGTGCAAGCCTCTTAAAAAAGGTTGAGCT-3’ |
